# Psychological determinants of self-management in migrants with chronic non-communicable diseases: self-efficacy and self-esteem

**DOI:** 10.3389/fpsyg.2026.1729089

**Published:** 2026-03-18

**Authors:** Karina Toro-Aguirre, Alfonso Urzúa, Neiber Maldonado-Suárez

**Affiliations:** 1 Escuela de Psicología, Universidad Católica del Norte, Antofagasta, Chile; 2 Centro Universitario del Sur, Universidad de Guadalajara, Guadalajara, Mexico

**Keywords:** chronic diseases, migration, self-efficacy, self-esteem, self-management

## Abstract

Within the framework of migration as a social determinant of health, it is important to study the psychological factors associated with self-management of chronic non-communicable diseases (CNCDs), a topic that has been little studied in south-south migrant populations. The present study aimed to analyze two mediation models. The first describes the effect of individual self-esteem as a mediator of the relationship between self-efficacy and self-management of CNCDs. The second model describes the effect of self-efficacy as a mediator in the relationship between self-esteem and self-management of CNCDs in South American migrants residing in Chile. A survey was conducted among 241 South-South migrants diagnosed with CNCDs and residing in five cities in Chile. Four *ad hoc* scales were applied to assess the study variables, analyzing the measurement models for each of them using the confirmatory factor analysis method. Subsequently, the hypothetical mediation model was evaluated using Structural Equation Modeling (SEM). The results show that self-efficacy has a positive and direct relationship with self-management of CNCDs, and self-esteem has a positive and direct relationship with self-management of CNCDs. Furthermore, both self-esteem and self-efficacy showed significant mediating effects in the association with self-management of CNCDs. While self-esteem acted as a relevant personal resource associated with self-efficacy, self-efficacy emerged as the more proximal and robust mediating mechanism linking self-esteem to self-management of CNCDs.

## 1 Introduction

Migration is a global social phenomenon. By 2024, 3.7% of the world’s population was living outside their country of origin (International Organization for Migration [IOM], 2024). The World Health Organization recognizes migration as a social determinant of health, as migrants are often exposed to a range of social and economic risk factors in destination countries that may negatively affect their health (World Health Organization [WHO], 2025). In this context, migrants may experience multiple conditions of social vulnerability including irregular migration status, limited access to basic services, family separation, reduced social support, socioeconomic disadvantage, poor housing conditions, unemployment or informal employment, and lower educational attainment that can negatively affect health literacy and the management of health-related behaviors. Moreover, interactions with the host society may involve experiences of discrimination, abuse, violence, or exploitation, all of which can directly impact migrants’ health and that of their family members (Davies et al., 2009; Spallek et al., 2011; Cabieses et al., 2013, 2018; World Health Organization [WHO], 2019, 2022a).

Health inequities are understood as gaps in the effective implementation of fundamental rights, whereby migration status in general, and irregular status in particular, increases the risk of adverse health outcomes (World Health Organization [WHO], 2008). These inequities and adverse conditions may manifest at both the individual and psychological levels, affecting both physical and mental well-being (Urzúa et al., 2017). During the migration process and throughout migrants’ stay in the host country, changes in living conditions, social environments, and lifestyles may expose individuals to new health risks and other social determinants of health. These changes may facilitate the adoption and maintenance of unhealthy behaviors, thereby increasing the risk of developing chronic non-communicable diseases (World Health Organization [WHO], 2021). Given their relevance to public health in countries, several studies have examined psychosocial and economic risk factors that may negatively affect migrants health, either maintaining or worsening health conditions, or contributing to the development of chronic non-communicable diseases (CNCDs) (Urzúa et al., 2017; International Organization for Migration [IOM], 2020; World Health Organization [WHO], 2022a,b). These health impacts can manifest themselves at the individual and psychological level, affecting both physical and mental well-being (Urzúa et al., 2017).

In a meta-analysis conducted by Nisar et al. (2023), they reported that the combined prevalence of diabetes was 9.0%, with a higher prevalence in North American countries (11.1%), while the combined prevalence of cardiovascular and respiratory diseases was 7.7%. They also observed significant heterogeneity among all studies, regardless of the disease. In South America, there are few studies on the prevalence of chronic non-communicable diseases (CNCDs) in the South-South migrant population. For example, in Venezuelan migrants, prevalences range from 9 to 14%, with the most common being hypertension, diabetes, asthma, arthritis, chronic pain, and mental disorders (Freier et al., 2021). In Chile, the overall prevalence of CNCDs among migrants is reported to be 6.41%, with diabetes standing out at 12.05% (Cabieses et al., 2022). In addition, it is reported that South American migrants experience delayed diagnosis and treatment of various CNCDs, such as hypertension, type II diabetes mellitus, cancer, and cardiovascular diseases (International Organization for Migration [IOM], 2017).

In this context, migrants with chronic non-communicable diseases may face specific challenges such as barriers to timely healthcare access, difficulties in treatment continuity, limited health literacy, financial constraints, and the need to adapt self-care behaviors within unfamiliar health systems and social environments (International Organization for Migration [IOM], 2020; World Health Organization [WHO], 2022a).

Considering the impact that CNCDs have on the health of migrants, it is essential that they have the capacity to manage them themselves, an action known as self-management. This is defined as the ability to cope with one or more chronic conditions in collaboration with family, community, and health professionals, encompassing behavioral, emotional, and clinical aspects with safety and confidence (Corbin and Strauss, 1988; Lorig and Holman, 2003; Schulman-Green et al., 2012). Among the psychological factors related to self-management in the general population, there are two variables that stand out for their strong relationship with the ability to manage the disease, and which are linked to a person’s perception of themselves: self-efficacy and self-esteem.

Self-efficacy stands out for its significant and positive relationship with self-management of CNCDs (Mann et al., 2009; Kong et al., 2019; Wilski et al., 2021; Chuang et al., 2021; Lai et al., 2021; Chen et al., 2022; Ni et al., 2022; Wang et al., 2022; Wu et al., 2022). According to Bandura (1997), self-efficacy is defined as individuals’ beliefs in their capabilities to organize and execute the courses of action required to manage prospective situations. In the healthcare context, self-efficacy can be understood as a person’s confidence in effectively managing their symptoms and adopting healthy behaviors (Wang et al., 2022). It is associated with better adherence to self-management behaviors and better health outcomes in the general population (Lai et al., 2021; Wilski et al., 2021; Ni et al., 2022; Wang et al., 2022). Despite the importance of studying this variable, there is a gap in research on the relationship between these variables in the migrant population.

Self-esteem, understood as the positive or negative assessment that a person has of themselves, is another relevant psychological factor in the self-management of chronic non-communicable diseases (CNCDs). Rosenberg (1965) defines it as a global attitude toward the self that includes beliefs and feelings about oneself. Low self-esteem can hinder self-management, while high self-esteem is associated with greater self-efficacy and, therefore, better management of CNCDs (Bedrov and Bulaj, 2018). Some studies suggest that self-efficacy mediates this relationship (Clark and Dodge, 1999; Flay et al., 2019). However, there is little evidence of a direct relationship between self-esteem and self-management of CNCDs. Thus, there is a gap in literature, especially in the migrant population.

From a theoretical standpoint, self-efficacy and self-esteem are closely related yet conceptually distinct constructs that represent different levels of self-perception (Bandura, 1997; Rosenberg, 1965; Judge et al., 2002). Self-esteem reflects a global and relatively stable evaluation of the self (Rosenberg, 1965), whereas self-efficacy refers to context-specific beliefs regarding one’s capacity to successfully perform behaviors required to achieve particular outcomes. This distinction is especially relevant in the context of chronic disease self-management, which involves concrete and sustained health-related behaviors that may depend more directly on perceived competence and confidence in one’s abilities than on global self-worth (Lorig and Holman, 2003; Schulman-Green et al., 2012). Social cognitive theory positions self-efficacy as a proximal determinant of health behaviors, suggesting that it may mediate the relationship between broader self-evaluative constructs and self-management outcomes (Bandura, 1997; Schwarzer and Fuchs, 1995). Conversely, other psychological models conceptualize self-esteem as a more distal personal resource that influences motivation, resilience, and confidence, potentially shaping self-efficacy beliefs over time (Rosenberg, 1979; Baumeister et al., 2003). Taken together, these perspectives indicate that different directional pathways between self-esteem, self-efficacy, and self-management are theoretically plausible, and supported by partial empirical evidence across different populations and health contexts (Flay et al., 2019).

Importantly, this theoretical ambiguity becomes particularly relevant in migrant populations. Migration-related stressors—such as socioeconomic instability, discrimination, acculturative stress, and barriers to healthcare access—may differentially affect global self-evaluations and task-specific confidence (Berry, 1997; Urzúa et al., 2017; World Health Organization [WHO], 2022a). These contextual factors may alter the relative contribution of self-esteem and self-efficacy to self-management behaviors, making it inappropriate to assume a single causal ordering between these variables. Therefore, comparing competing mediation models allows for a more nuanced understanding of the psychological mechanisms underlying self-management of chronic diseases in South-South migrant populations.

This study falls within the still largely unexplored field of South-South migration, that is, migrants who move from one country to another within the South American region. This type of migration has experienced a notable increase, with estimates suggesting that by 2024 there will be approximately 10 million migrants in the region, 80% of whom will be intra-regional (International Organization for Migration [IOM], 2024).

Considering that migration involves multiple social and economic challenges that can affect health, the study of psychological variables such as self-efficacy and self-esteem becomes crucial to understanding how migrants cope with CNCDs, so that they can maintain adherence to treatments and healthy habits despite the barriers they face. Clarifying the directionality of the mediation between self-esteem and self-efficacy has both theoretical and practical relevance. From a theoretical perspective, identifying whether self-efficacy mediates the relationship between self-esteem and self-management, or vice versa, contributes to refining psychological models of self-regulation in health contexts by distinguishing between proximal and distal mechanisms of action. From a practical standpoint, this distinction has direct implications for intervention design, as it informs whether self-management programs should primarily focus on strengthening task-specific confidence (self-efficacy), enhancing global self-worth (self-esteem), or addressing both sequentially. This question is particularly relevant for migrant populations in Chile, where structural barriers to healthcare, experiences of discrimination, and socioeconomic vulnerability may undermine either global self-evaluations or confidence in managing health conditions. Determining the predominant mediation pathway can guide the development of culturally and contextually tailored interventions aimed at improving chronic disease self-management among migrants, thereby addressing an important public health challenge in the Chilean context.

Assuming that both self-efficacy and self-esteem will have a direct effect on self-management, we have chosen two models to test, seeking to deepen our understanding of how these psychological processes influence self-management. Specifically, the present study aims to empirically compare two theoretical mediation models explaining the relationship between self-efficacy, self-esteem, and self-management of chronic diseases in migrant populations.

In the first model, it is hypothesized that self-esteem mediates the relationship between self-efficacy and self-management, such that higher self-efficacy is associated with higher self-esteem, which in turn is associated with better self-management.

In the second model, it is hypothesized that self-efficacy mediates the relationship between self-esteem and self-management, such that higher self-esteem is associated with greater self-efficacy, which in turn is associated with better self-management.

**FIGURE 1:**
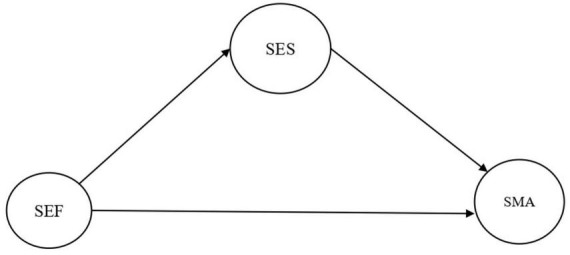
Model 1. SEF, self-efficacy; SES, self-esteem; SMA, self-management.

**FIGURE 2:**
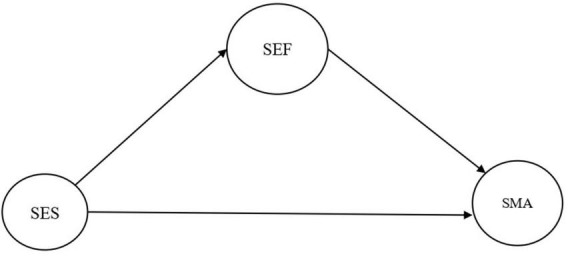
Model 2. SEF, self-efficacy; SES, self-esteem; SMA, self-management.

The models to be tested can be seen in Figures 1, 2.

## 2 Materials and methods

A non-experimental, cross-sectional design was used. Although this design does not allow causal inference, it is appropriate for examining theoretically specified associations and indirect effects among psychological constructs using correlational and structural equation modeling approaches (Ato et al., 2013; Coolican, 2018).

### 2.1 Participants

Questionnaires were administered to South-South migrants residing in the cities of Arica, Antofagasta, Santiago, Concepcion, and Temuco, Chile. Data collection took place between 2024 and 2025. Participants had to meet the following inclusion criteria: (1) be over 18 years of age; (2) have resided in Chile for more than 3 months; (3) be from South American countries; and (4) have a diagnosis of chronic non-communicable disease (CNCD).

Given the impossibility of obtaining the exact number of migrants in Chile, participants were recruited using a non-probabilistic purposive sampling method combined with a snowball sampling technique, whereby each participant had the opportunity to refer other migrants who met the inclusion criteria for the general study to the researcher. Data were collected from 1,844 participants, who were invited to participate in health centers, migrant organizations, places with a high influx of migrants, and housing sectors with a high migrant population. Of the total number of respondents, only 247 met the criterion of having an official diagnosis of chronic disease, as expressed through self-reporting.

Prior to analysis, missing data patterns were examined. Five cases were excluded due to having more than 20% missing responses across the study variables, and one additional case was removed because the country of origin was not specified. Among the remaining 241 participants, descriptive inspection of all items from the self-efficacy, self-esteem, and self-management instruments indicated complete data, with no missing values. Therefore, no imputation procedures were required for the final analytical sample (Table 1).

**TABLE 1:** Sociodemographic characteristics of the participants.

Variable	Category	Frequency	%
Age (years)	*M* = 45.02, *SD* = 13.27	–	–
Sex	Male	84	34.9
	Female	157	65.1
Monthly household income (CLP)	Less than $100,000	27	11.2
	Between $100,000 and 300,000	47	19.5
	Between $300,000 and 600,000	93	38.6
	Between $600,000 and 1,000,000	46	19.1
	Between $1,000,000 and 1,500,000	19	7.9
	More than $1,500,000	9	3.7
Occupation/activity	No response	3	1.2
	Employed	175	72.6
	Retired	8	3.3
	Unemployed	32	13.3
	Homemaker	20	8.3
	Student	3	1.2
Educational attainment	No formal education	18	7.5
	Primary education	42	17.4
	Secondary education	75	31.1
	Incomplete technical studies	11	4.6
	Partial university studies (diplomas, unfinished programs)	44	18.2
	Completed university studies	39	16.2
	Postgraduate studies	12	5.0
Country of origin	Colombia	85	35.3
	Peru	66	27.4
	Venezuela	71	29.5
	Bolivia	19	7.9
Chronic non-communicable diseases (CNDs)	Diabetes	58	24.1
	Hypertension	106	44.0
	Diabetes and hypertension	27	11.2
	Respiratory diseases	26	10.8
	Musculoskeletal diseases	7	2.9
	Endocrine diseases	13	5.4
	Cancer	4	1.7

Monthly household income is reported in Chilean pesos (CLP). At October 12, 2025, 1 USD ≈ 961.54 CLP.

### 2.2 Procedures

This study is part of a larger project funded by Fondecyt Regular No. 1230164 on Health Promotion in Migrant Populations. The study strictly complies with the bioethical codes applicable to research involving humans. The protocol was approved by the Scientific Ethics Committee (CEC-UCN) (res 15/03/2023), ensuring the use of informed consent and the protection of privacy and data confidentiality. Before collecting data, researchers informed participants about the study’s objective and methodology, obtaining their prior consent in person. Information was collected using printed questionnaires. Given the characteristics of migrant populations, particularly those living with chronic conditions, probabilistic sampling strategies are often not feasible due to the absence of reliable sampling frames, high mobility, and structural barriers to participation. Therefore, a non-probabilistic sampling strategy combining purposive and snowball sampling was employed. This approach is widely recommended for research involving migrant and other hard-to-reach or socially vulnerable populations. Importantly, the primary aim of the present study was not to estimate population prevalence, but to examine theoretically grounded relationships between psychological constructs. In this context, non-probabilistic sampling is considered methodologically appropriate for testing relational and explanatory models in psychological research (Atkinson and Flint, 2001; Heckathorn, 1997; Tyldum and Johnston, 2014).

The interviews were conducted mainly in public institutions and organizations linked to migrants, such as the Jesuit Migrant Service, the International Organization for Migration, the Chilean Catholic Institute of Migration, the Migrant Office of the Municipality of Antofagasta, the Colombian Consulate, the Pastoral Care of the Catholic University of the North, as well as their workplaces, health centers, camps, and neighborhoods with high concentrations of migrants, among others.

### 2.3 Measures

#### 2.3.1 Demographic and clinical characteristics

Questions were included on sociodemographic factors such as age, sex, country of origin, length of residence in Chile, income, occupation/activity, educational level and clinical factors such as diagnoses of chronic diseases (diabetes, hypertension, others), time of diagnosis, medical treatment and attendance to chronic control.

#### 2.3.2 Self-management

To assess self-management of NCD, we applied the Spanish adaptation (Peñarrieta-de Córdova et al., 2014) of the Partners in Health Scale (PHS) (Battersby et al., 2003). The scale consists of 12 items with a continuous rating scale ranging from 0 to 8. This instrument allows the assessment of behavior globally and in three dimensions: (1) knowledge about their health and disease, (2) management of the physical, emotional and social impact derived from their disease, and (3) the dimension of adherence understood as adherence to medical treatment, level of communication with the physician and health care providers. Its interpretation is the higher the score, the better the self-management (Peñarrieta-de Córdova et al., 2014). In similar populations, a Cronbach’s alpha of 0.88 has been reported for the scale in the Mexican context and 0.85 in the older adult population in Peru (Peñarrieta-de Cordova et al., 2020).

#### 2.3.3 Self-efficacy

To assess perceived general self-efficacy we used the “Measurement of perceived self-efficacy” scale by Schwarzer adapted to Spanish by Baessler and Shwarzer (1996) and validated in Chile by Cid et al. (2010). The scale is composed of 10 items that make up the Likert-type scale of General Self-Efficacy, with a minimum score of 10 points and a maximum score of 40 points. The person responds to each item according to what he or she perceives of his or her ability at the moment, from incorrect (1 point), barely true (2 points), well true (3 points) or true (4 points) (Baessler and Schwarzer, 1996; Schwarzer and Jerusalem, 1997). In this scale, the higher the score, the higher the perceived general self-efficacy. This instrument has been validated in different South American countries such as Peru with a Cronbach’s alpha of 0.75 (Grimaldo, 2005) in Chile with a Cronbach’s alpha of 0.84 (Cid et al., 2010), Colombia with a Cronbach’s alpha of 0.83 and 0.74 (Juárez and Contreras, 2008; Escobar and Zambrano, 2015) and Venezuela with a Cronbach’s alpha of 0.92 (García-Álvarez et al., 2022) demonstrating adequate reliability.

#### 2.3.4 Self-esteem

The Rosenberg Self-Esteem Scale (RSES) developed by Morris Rosenberg in 1965 was used, translated and adapted into various languages such as Portuguese, Chinese, Italian, Spanish, among others. It is aimed at adolescents, adults, and older adults with the objective of assessing and identifying self-esteem (Rojas-Barahona et al., 2009; Delgado and Ortiz, 2021). After conducting various studies in more than 50 countries with different cultures, it was determined that the EAR has an internal consistency of 0.87 Cronbach’s alpha and a reliability of 0.75. It consists of 10 statements with Likert-type responses with scores ranging from 1 to 4, depending on the degree of agreement with the statements. The score range is between 10 (low self-esteem) and 40 (high self-esteem) (Lima and de Souza, 2019; Bueno-Pacheco et al., 2020; Vilca et al., 2022).

### 2.4 Data analysis

In the participants section, the sociodemographic characteristics of the participants were presented, followed by descriptive statistics for each of the scales used in the study. Prior to the structural analyses, the measurement properties of all instruments were examined using confirmatory factor analysis (CFA) with an ordinal estimation approach (WLSMV) (Holtmann et al., 2016; Ximénez et al., 2022). Model fit was assessed using complementary indices, including CFI (McDonald and Marsh, 1990), TLI (Bollen, 1989), RMSEA (Browne and Cudeck, 1992), and SRMR (Shi et al., 2022).

In line with current recommendations for ordinal CFA, greater weight was given to incremental (CFI, TLI) and residual-based (SRMR) indices, given the known sensitivity of RMSEA under WLSMV estimation. Model re-specifications, when required, were theoretically justified based on item content similarity and prior psychometric evidence. Only measurement models showing acceptable fit were retained for subsequent structural equation modeling. Internal consistency was assessed using McDonald’s omega (ω), given its suitability for latent variable models and ordinal indicators (Kalkbrenner, 2023; McDonald, 1999).

Prior to the main analyses, statistical assumptions were examined. Univariate normality was assessed and not confirmed; therefore, robust estimation methods were employed for the structural equation modeling analyses, specifically the weighted least squares mean and variance adjusted estimator (WLSMV), which is appropriate for ordinal data and non-normal distributions. Linearity was evaluated through visual inspection of scatterplots, partial regression plots, and plots of standardized residuals against fitted values. The residuals were randomly distributed around zero, with no evidence of systematic curvature or non-linear patterns, and partial regression plots indicated approximately linear relationships between predictors and the outcome variable. Multicollinearity was examined using tolerance and variance inflation factor (VIF) statistics, as well as collinearity diagnostics based on condition indices. All predictors showed tolerance values above 0.80 and VIF values close to 1, indicating no evidence of multicollinearity. In addition, condition indices were below critical thresholds and variance proportions did not suggest problematic shared variance among be related toors. Given the ordinal nature of the response scale, all observed values fell within the theoretical response range. Influence diagnostics using Cook’s distance, leverage values, and Mahalanobis distance did not identify any influential cases, suggesting the absence of problematic univariate or multivariate outliers. Finally, independence of observations was ensured by study design, as each participant contributed a single set of responses and the data did not present a hierarchical or nested structure.

Subsequently, the means, standard deviations, and Pearson correlation coefficients between the variables included in the mediation model were calculated. To examine the proposed mediation hypotheses, two alternative structural equation models (SEM) were tested using the same measurement structure. In both models, self-efficacy, self-esteem, and self-management of chronic non-communicable diseases were specified as latent variables measured by their respective items. The models differed only in the direction of the mediation pathway: in the first model, self-esteem was specified as a mediator between self-efficacy and self-management, whereas in the second model self-efficacy was specified as a mediator between self-esteem and self-management. Models were estimated using robust methods appropriate for ordinal indicators, and model fit was evaluated using multiple complementary indices (CFI, TLI, RMSEA, and SRMR). Given that both models shared identical measurement specifications, model comparison was based on explained variance (*R*^2^) in self-management and on the magnitude and coherence of the structural paths, rather than on global fit indices alone (Dominguez-Lara, 2019; Marsh and Hocevar, 1985; Ximénez et al., 2022).

All reliability, correlation, and structural equation modeling analyses were conducted in the R Studio statistical environment, using the psych, skimr, lavaan, and Hmisc packages.

## 3 Results

### 3.1 Measurement models

The unifactorial CFA model for self-efficacy showed excellent incremental fit (CFI = 0.974, TLI = 0.966) and low residual-based misfit (SRMR = 0.049). Although RMSEA was elevated (RMSEA = 0.166), the overall pattern of fit indices and the absence of substantial localized misfit supported the adequacy of the measurement model in this migrant sample.

For self-esteem, the initial unifactorial model showed inadequate global fit. A revised model allowing correlated residuals between three pairs of items with highly similar wording (SES01–SES02, SES07–SES08, SES09–SES10) resulted in a substantial improvement in model fit (CFI = 0.937, TLI = 0.911). This specification is consistent with well-documented method effects associated with item wording in the Rosenberg Self-Esteem Scale (Gnambs and Schroeders, 2017). Despite a persistently elevated RMSEA, the revised model was considered acceptable based on the convergence of incremental and residual-based indices.

The unifactorial model for self-management of chronic non-communicable diseases (CNCDs) demonstrated good fit to the data (CFI = 0.958, TLI = 0.945; SRMR = 0.045). Correlated residuals were specified between three pairs of adjacent items with overlapping content (SMA01–SMA02; SMA04–SMA05; SMA10–SMA11), capturing local dependencies without altering the underlying factorial structure. As in the previous models, RMSEA values were interpreted cautiously due to the ordinal estimation method.

Overall, the measurement models showed adequate structural validity, supporting the use of the latent constructs in subsequent structural analyses. The internal consistency of the instruments was assessed using McDonald’s omega (ω) (Table 2).

**TABLE 2:** The internal consistency of the instruments.

Scale	Number of items	McDonald’s ω
Self-efficacy	10	0.94
Self-esteem	10	0.72
Self-management	12	0.94

Internal consistency was assessed using McDonald’s omega (ω), which provides a more accurate reliability estimate under congeneric measurement models.

The instruments showed adequate levels of reliability: self-efficacy (α = 0.94), self-esteem (α = 0.76), and chronic disease self-management (α = 0.94). These results indicate internal consistency ranging from acceptable to excellent across the scales used.

### 3.2 Correlation analysis

Pearson correlations were explored between sociodemographic variables (age, income, and educational level) and psychological variables (self-efficacy, self-esteem, and self-management of chronic non-communicable diseases). The results are presented in Table 3.

**TABLE 3:** Correlations between variables.

Variable	1	2	3	4	5	6
1. Age (years)	–	–	–	–	–	–
2. Income	0.00					
3. Education	−0.10	0.51**				
4. Self-efficacy	0.09	0.14*	0.13*			
5. Ethnic identity	0.04	−0.07	−0.07	0.18**		
6. Self-esteem	0.10	0.24**	0.27**	0.39**	0.07	
7. Self-management	0.23**	0.18**	0.27**	0.44**	0.17**	0.40**

*p < 05;

**p < 0.01.

Age showed a positive and significant correlation with chronic disease self-management (*r* = 0.23, *p* < 0.001), although it did not present significant associations with self-efficacy or self-esteem. Monthly income was positively correlated with educational level (*r* = 0.51, *p* < 0.001), self-efficacy (*r* = 0.14, *p* = 0.030), self-esteem (*r* = 0.24, *p* < 0.001), and self-management (*r* = 0.18, *p* = 0.005). Educational level showed positive and significant correlations with self-efficacy (*r* = 0.13, *p* = 0.042), self-esteem (*r* = 0.27, *p* < 0.001), and NCD self-management (*r* = 0.27, *p* < 0.001). Regarding psychological variables, self-efficacy was positively related to self-esteem (*r* = 0.39, *p* < 0.001) and NCD self-management (*r* = 0.44, *p* < 0.001). Finally, self-esteem showed significant positive correlations with self-management (*r* = 0.40, *p* < 0.001), as well as with self-efficacy and educational level.

### 3.3 SEM mediation models

The factorial structure of the latent variables included in the SEM mediation models was examined using CFA. Standardized factor loadings, standard errors, and confidence intervals for all indicators are reported in Table 4. All factor loadings were statistically significant (*p* < 0.001), supporting the adequacy of the measurement model.

**TABLE 4:** Factorial structure of the latent variables.

Latent	Items	λ	SE	*z*	*p*	IC 95%
Self-efficacy	SEF01	0.65	0.04	15.94	***	[0.57, 0.73]
	SEF02	0.82	0.03	29.59	***	[0.76, 0.87]
	SEF03	0.80	0.03	26.93	***	[0.74, 0.86]
	SEF04	0.84	0.02	36.42	***	[0.80, 0.89]
	SEF05	0.90	0.02	57.30	***	[0.87, 0.93]
	SEF06	0.86	0.02	40.87	***	[0.82, 0.90]
	SEF07	0.87	0.02	47.60	***	[0.83, 0.91]
	SEF08	0.91	0.02	61.53	***	[0.89, 0.94]
	SEF09	0.92	0.01	67.75	***	[0.89, 0.94]
	SEF10	0.93	0.01	67.25	***	[0.90, 0.95]
Self-esteem	SES01	0.71	0.04	16.52	***	[0.63, 0.80]
	SES02	0.67	0.04	16.25	***	[0.59, 0.75]
	SES03	0.60	0.05	11.96	***	[0.50, 0.69]
	SES04	0.67	0.05	14.14	***	[0.58, 0.76]
	SES05	0.34	0.07	5.07	***	[0.21, 0.47]
	SES06	0.86	0.03	33.96	***	[0.81, 0.91]
	SES07	0.87	0.02	37.21	***	[0.82, 0.92]
	SES08	0.26	0.07	3.94	***	[0.13, 0.39]
	SES09	0.69	0.04	16.31	***	[0.60, 0.77]
	SES10	0.80	0.04	22.05	***	[0.73, 0.87]
Self-management	SMA01	0.85	0.02	49.21	***	[0.82, 0.89]
	SMA02	0.89	0.02	53.17	***	[0.85, 0.92]
	SMA03	0.87	0.02	51.72	***	[0.84, 0.90]
	SMA04	0.80	0.02	35.10	***	[0.76, 0.85]
	SMA05	0.71	0.03	24.26	***	[0.65, 0.76]
	SMA06	0.81	0.02	36.56	***	[0.76, 0.85]
	SMA07	0.87	0.02	52.00	***	[0.84, 0.91]
	SMA08	0.85	0.02	46.00	***	[0.82, 0.89]
	SMA09	0.80	0.02	35.25	***	[0.75, 0.84]
	SMA10	0.84	0.02	46.83	***	[0.81, 0.88]
	SMA11	0.86	0.02	47.91	***	[0.82, 0.90]
	SMA12	0.75	0.03	27.60	***	[0.70, 0.80]

λ, standardized factor loading; SE, standard error. CFA estimated using WLSMV. All factor loadings were statistically significant (***p < 0.001). Both SEM models share the same measurement model; therefore, factor loadings are identical across models. Although two self-esteem items showed lower standardized loadings (SES05, SES08), they were retained based on their theoretical relevance and the overall adequacy of the measurement model.

Two alternative mediation models were tested using SEM, each specifying identical measurement structures but differing in the direction of the mediation pathway. Both models assessed the direct, indirect, and total effects, as well as the variance explained by the full model (Table 5).

**TABLE 5:** Comparison of two SEM mediation models between self-efficacy, self-esteem, and self-management of chronic diseases.

Effect	Model 1					Model 2				
	β	SE	*z*	*p*	IC 95%	β	SE	*z*	*p*	IC 95%
a	0.40	0.06	7.39	***	[0.30, 0.51]	0.40	0.06	7.39	***	[0.30, 0.51]
b	0.24	0.06	4.08	***	[0.12, 0.35]	0.38	0.06	6.79	***	[0.27, 0.49]
c1	0.38	0.06	6.79	***	[0.27, 0.49]	0.24	0.06	4.08	***	[0.12, 0.35]
Ab	0.10	0.03	3.54	***	[0.04, 0.15]	0.15	0.03	4.84	***	[0.09, 0.25]
C	0.47	0.05	10.01	***	[0.38, 0.57]	0.39	0.05	7.51	***	[0.29, 0.49]

a, effect of the independent variable on the mediator; b, effect of the mediator on the dependent variable; c1, direct effect of the independent variable on the dependent variable controlling for the mediator; Ab, indirect effect; C, total effect. All coefficients are standardized estimates (β) obtained from structural equation modeling. Confidence intervals correspond to 95% robust confidence intervals.

***p < 0.001.

#### 3.3.1 Model 1: self-efficacy, self-esteem, self-management

**FIGURE 3:**
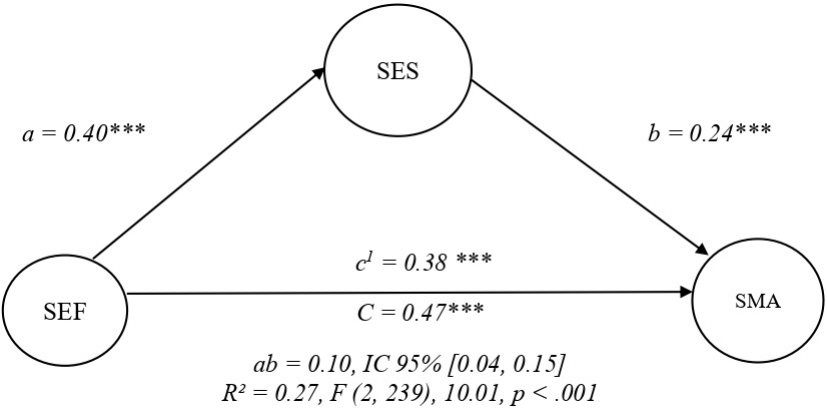
Mediation model between self-efficacy, self-esteem, and self-management. SEF, self-efficacy; SES, self-esteem; SMA, self-management. Observed indicators are included in the SEM analyses but are not displayed in the figure for clarity. ***p < 0.001.

In the first mediation model, self-esteem was specified as a mediator between self-efficacy and self-management of chronic non-communicable diseases. The model showed good fit to the data (CFI = 0.97, TLI = 0.96, RMSEA = 0.07, SRMR = 0.08) and explained 27% of the variance in self-management. Self-efficacy had a significant positive effect on self-esteem (β = 0.40, *p* < 0.001), and self-esteem was positively associated with self-management (β = 0.24, *p* < 0.001). In addition, the direct effect of self-efficacy on self-management remained significant (β = 0.38, *p* < 0.001), indicating partial mediation. The indirect effect of self-efficacy on self-management through self-esteem was also significant (β = 0.10, *p* < 0.001), supporting the mediating role of self-esteem in this model (Figure 3).

#### 3.3.2 Model 2: self-esteem, self-efficacy, self-management

**FIGURE 4:**
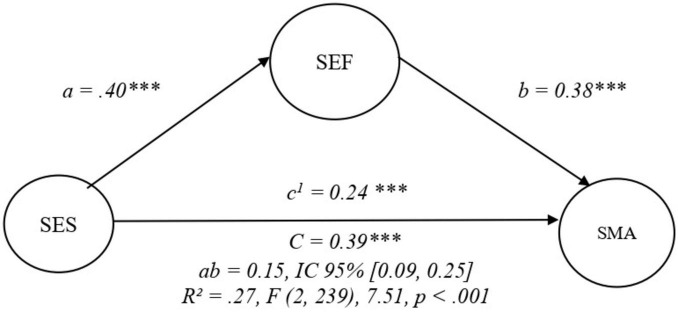
Mediation model between self-esteem, self-efficacy, and self-management. Observed indicators are included in the SEM analyses but are not displayed in the figure for clarity. ***p < 0.001.

In the second mediation model, self-efficacy was specified as a mediator between self-esteem and self-management. This model showed identical global fit indices (CFI = 0.97, TLI = 0.96, RMSEA = 0.07, SRMR = 0.08) and explained the same proportion of variance in self-management (*R*^2^ = 0.27). Self-esteem was positively associated with self-efficacy (β = 0.40, *p* < 0.001), and self-efficacy showed a strong positive effect on self-management (β = 0.38, *p* < 0.001). The direct effect of self-esteem on self-management remained significant (β = 0.24, *p* < 0.001), indicating partial mediation. The indirect effect of self-esteem on self-management through self-efficacy was also significant (β = 0.15, *p* < 0.001), supporting self-efficacy as a mediating mechanism in the relationship between self-esteem and self-management (Figure 4).

Although both mediation models showed identical global fit indices and explained the same proportion of variance in self-management (*R*^2^ = 0.27), recent methodological work has emphasized that, particularly in SEM models with small degrees of freedom, global fit indices should not be used as the sole basis for model selection and must be interpreted in light of the model’s structure and theoretical coherence.(Shi et al., 2022). Accordingly, the preference for Model 2 was based on three converging criteria: (a) the magnitude and coherence of the structural effects, (b) the proportion of the total effect accounted for by the mediating pathway (VAF), and (c) theoretical parsimony. First, regarding the magnitude and coherence of the structural effects, Model 2 displayed a clearer hierarchical pattern, in which self-esteem was strongly associated with self-efficacy (β = 0.40, *p* < 0.001), and self-efficacy emerged as the strongest proximal predictor of self-management (β = 0.38, *p* < 0.001), while the direct effect of self-esteem on self-management was comparatively smaller (β = 0.24, *p* < 0.001). This configuration is consistent with sociocognitive models that conceptualize self-efficacy as a more immediate determinant of health-related behaviors. Second, the proportion of the total effect accounted for by the indirect pathway was substantially larger in Model 2, with 38.5% of the total effect mediated through self-efficacy, compared to about 21.3% in Model 1, indicating a more central mediating role in the second model (Nitzl et al., 2016). Finally, Model 2 was considered more parsimonious from a theoretical standpoint, as it clearly distinguishes between self-esteem as a distal personal resource and self-efficacy as a proximal mechanism directly linked to self-management behaviors. Taken together, these converging structural, mediational, and theoretical considerations support the interpretation of self-efficacy as the primary mediating mechanism linking self-esteem to self-management of chronic non-communicable diseases in migrant populations.

## 4 Discussion

The findings of this study support the importance of the psychological variables of self-efficacy and self-esteem in the self-management of chronic non-communicable diseases (CNCDs) in migrants. The high reliability of the instruments used supports the validity of the measurements, while the correlations and multiple mediation models offer a more accurate understanding of the relationships between these variables.

Both self-efficacy and self-esteem were significantly and positively associated with self-management, which is in line with previous literature (Ozturk et al., 2022; Huang et al., 2024; Wilski and Tasiemski, 2016; Li et al., 2025). Self-esteem also showed a significant relationship with self-efficacy, supporting theories that a positive self-image may be associated with belief in one’s ability to act effectively (Bonsaksen et al., 2015; Nasiriziba et al., 2020; Ozturk et al., 2022). In migrant populations, these associations should be interpreted in light of migrant-specific barriers to self-management, such as limited access to healthcare services, language barriers, precarious employment, and experiences of discrimination within host health systems. Nonetheless, all variables were assessed through self-report measures, which may be subject to social desirability or recall bias, potentially influencing the strength of the associations observed.

The analyses showed that sociodemographic variables such as educational level and income are positively associated with psychological variables and self-management, which is consistent with previous research. Several studies have shown that sociodemographic variables such as educational level and income are positively associated with self-efficacy and self-esteem, which in turn may be associated with better self-management of chronic diseases. For example, Bo et al. (2021) found that higher educational levels are related to greater self-efficacy, being associated with effective self-management behaviors in people with type 2 diabetes. Similarly, Sullivan et al. (2021) reported that higher income be related to greater self-efficacy for chronic pain management, which has been associated with better self-management outcomes.

Both mediation models were significant, with Model 1 highlighting the mediating role of self-esteem in the relationship between self-efficacy and self-management of CNCDs. Although both mediation models were significant, Model 2 presents more robust results, with self-esteem theoretically positioned in relation to self-efficacy, which in turn be associated with self-management of chronic diseases. Given the cross-sectional design, these mediation pathways should be understood as theoretically informed rather than empirically causal. These pathways may also shaped by contextual stressors linked to migration, including socioeconomic instability, reduced social support, and barriers to continuity of care. Longitudinal studies are needed to confirm the temporal ordering of these psychological processes.

This suggests that higher levels of self-esteem could be linked to higher levels of self-efficacy and be associated with self-management of CNCDs. This finding is particularly relevant in contexts of vulnerability such as migration, where self-esteem may be affected by discrimination, loss of social status, structural barriers, and other social determinants (Urzúa et al., 2021). Previous studies have found that self-esteem may function as a personal resource related to self-efficacy, being associated with adherence and self-management behaviors (Schwarzer and Fuchs, 1995; Schunk and DiBenedetto, 2020). Therefore, interventions oriented toward promoting self-esteem could represent a promising avenue for supporting self-efficacy and, consequently, self-management of the disease, especially in the south-south migrant population. Such interventions may benefit from being culturally sensitive and context-aware, incorporating components that address health literacy, familiarity with healthcare systems, and access-related barriers commonly faced by migrants. Nevertheless, the heterogeneity of chronic diseases included in this study, analyzed as a single category, may mask condition-specific self-management processes. Future research should examine these relationships across specific chronic conditions and diseases trajectories. This invites us to consider comprehensive interventions that strengthen both individual resources and socio-community protective factors, targeting clinical psychological health interventions focused on self-esteem that could be relevant for the health of migrants, especially in the self-management of CNCDs.

Some limitations should be acknowledged in this study. The findings should be interpreted with caution given the cross-sectional nature of the design, which does not allow for causal inferences regarding the directionality of the observed relationships.

The sample consisted exclusively of South-South migrants residing in Chile, which may limit the generalizability of the findings to other migrant populations, countries, or migration contexts. Migratory experiences and structural conditions vary widely across regions, and the observed relationships may differ in other settings. Despite the aforementioned limitations, this study contributes to the literature by providing a theoretically grounded comparison of alternative mediation models in an underexplored population, offering relevant implications for culturally and contextually tailored interventions in migrant health.

## 5 Conclusion

This study provides empirical evidence supporting the relevance of self-esteem and self-efficacy as key psychological factors associated with the self-management of CNCDs in migrants. Both mediation models show that these variables are not only related to self-management, but also interact significantly, suggesting indirect routes of influence. It was identified that self-esteem may be related to self-efficacy, being associated with higher levels of perceived ability to manage one’s own health. This has relevant implications for the design of psychoeducational, community-based, and social determinants of health interventions, which should include components aimed at reinforcing self-esteem and self-efficacy beliefs as potentially relevant components associated with chronic disease management in migrant populations. Future studies could explore these models in different migrant groups, incorporate cultural or structural variables, and longitudinally evaluate the effects of psychological interventions on these variables and their impact on physical and emotional health.

## Data Availability

The raw data supporting the conclusions of this article will be made available by the authors, without undue reservation.
